# A thin film efficient pn-junction thermoelectric device fabricated by self-align shadow mask

**DOI:** 10.1038/s41598-020-57991-y

**Published:** 2020-01-23

**Authors:** Gilbert Kogo, Bo Xiao, Samuel Danquah, Harold Lee, Julien Niyogushima, Kelsea Yarbrough, Aaditya Candadai, Amy Marconnet, Sangram K. Pradhan, Messaoud Bahoura

**Affiliations:** 1 Center for Materials Research, Norfolk State University, Norfolk, Virginia 23504 USA; 2 Mechanical Engineering Purdue University, West Lafayette, In 47907 USA; 3 Department of Engineering, Norfolk State University, Norfolk, Virginia 23504 USA

**Keywords:** Environmental impact, Devices for energy harvesting

## Abstract

Large area highly crystalline MoS_2_ and WS_2_ thin films were successfully grown on different substrates using radio-frequency magnetron sputtering technique. Structural, morphological and thermoelectric transport properties of MoS_2,_ and WS_2_ thin films have been investigated systematically to fabricate high-efficient thermal energy harvesting devices. X-ray diffraction data revealed that crystallites of MoS_2_ and WS_2_ films are highly oriented in 002 plane with uniform grain size distribution confirmed through atomic force microscopy study. Surface roughness increases with substrate temperature and it plays a big role in electron and phonon scattering. Interestingly, MoS_2_ films also display low thermal conductivity at room temperature and strongly favors achievement of higher thermoelectric figure of merit value of up to 1.98. Raman spectroscopy data shows two distinct MoS_2_ vibrational modes at 380 cm^−1^ for E^1^_2g_ and 410 cm^−1^ for A_1g_. Thermoelectric transport studies further demonstrated that MoS_2_ films show p-type thermoelectric characteristics, while WS_2_ is an n-type material. We demonstrated high efficient pn-junction thermoelectric generator device for waste heat recovery and cooling applications.

## Introduction

Global energy consumption is increasing day by day due to industrial and high population growth. There is a major global challenge in striving to meet the future energy demand in renewable, sustainability, clean, and safe energy sources^1,2^. One way in improving the electricity demand is by scavenging waste heat using thermoelectric generators^3^. The conversion of waste heat into electricity will provide alternative energy that will reduce our dependence on fossil fuels and reduce greenhouse gas emissions^4^. The performance of thermoelectric energy conversion devices depends on thermoelectric figure of merit ZT (Eq. 1), where S, σ, *κ*_*total*_ = *κ*_*e*_ + *κ*_*l*_, and T are the Seebeck coefficient, electrical conductivity, total thermal conductivity, and absolute temperature, respectively^5^. Thermal conductivity comes from two sources: (1) electrons and holes transporting heat *κ*_*e*_ and (2) phonons travelling through the lattice *κ*_*l*_^6^.

1 $$ZT=\frac{{S}^{2}\sigma }{{k}_{total}}$$

According to the figure of merit, good thermoelectric materials should possess high *S* value to have high voltage output, high *σ* value to reduce Joule heating, and low *κ* value to maintain large temperature difference^2^. Among the four quantities in Eq. (1) above, *S, σ*, and κ_e_ are interrelated to the electronic structure of the material, and κ_*l*_ is mainly related to the lattice vibration^7^. One possible way to improve the figure of merit is to reduce the lattice thermal conductivity without significantly altering the electronic properties of the material^8^.

The Seebeck coefficient is mostly considered as a bulk materials property. The figure of merit for most efficient bulk semiconductor (Bi_2_Te_3_, Sb_2_Te_3_) based device has been stable over the past several decades, with ZT value close to 1 and efficiency is about 10% of the Carnot limit. By reducing the size of the materials from 3D to lower dimension, the effect of interface becomes more significant as the hot and cold junctions are closer to each other and therefore the Seebeck coefficient is not independent of size anymore. Theoretical study proposed by other group shows that the reduced dimensionality of heterostructure/superlattice could be used to enhance the electronic density of state^9–12^. Hence, the mean free path of electron is longer in thin films compared to the one in the bulk material. Consequently, the electrons energies remain high because they do not get sufficient time to thermalize with the lattice.

Currently, the possibility of tailoring thermal and electrical properties offered by the heterostructure materials has attracted considerable attention. The strategy of rationally engineering semiconductor interfaces could enhance the ZT in thermoelectric (TE) materials with heterostructures. In these heterostructures, the significant enhancement performance is believed to result from the growth by preferential scattering of low-energy carriers more effectively than high ones and the reduction of the thermal conduction via scattering phonons at the heterostructured interfaces^13–15^. So far, the maximum reported room-temperature *ZT* to date, ~2.4 by Venkatasubramanian *et al*., was reached using superlattice heterostructure made up of *p*-type Bi_2_Te_3_/Sb_2_Te_3_ and was attributed to good electrical transport and very low thermal conductivity^16^. Although, Bi_2_Te_3_ and Sb_2_Te_3_ are more promising materials and the device made out of it are showing highest figure of merit, but they are very toxic, carcinogenic in nature and are not bio friendly.

As an alternative bio-friendly materials, the transition metal dichalcogenides recently shows very promising waste heat conversion performance by limiting its thickness to one layer or multiple layers and, more attentions are given to MoS_2_, MoSe_2_, WS_2_, WSe_2_ and so on^17–21^. Among these materials, the electronic band structure of monolayer or bi layers thickness of MoS_2_ and WS_2_ show an excellent carrier mobility compare to the bulk and exhibit the band tunability from indirect to direct band gap semiconductor^22^. Hence, the electronic device made out of these materials shows exceptional device performance and these nano sheet are very good thermal insulator and favor to prevent the heat flow during operation^23–25^. Similarly, previous study based on theoretical calculation emphasized that the single layer of WS_2_ and MoS_2_ films also show higher value of thermoelectric figure of merit known as ZT^18,19,21^. The high figure of merit in few layers of MoS_2_/WS_2_ heterostructure are considered as one of the potential thermoelectric materials for optimum waste heat energy harvesting.

The thermoelectric device performance and its waste heat conversion efficiency can be derived from ZT and Carnot efficiency using the following equation as below^2,26–28^.

2 $${\eta }_{TEG}=\left(\frac{{T}_{h}-{T}_{c}}{{T}_{h}}\right)\left[\frac{\sqrt{1+Z{T}_{M}}-1}{\sqrt{1+Z{T}_{M}}+\frac{{T}_{c}}{{T}_{h}}}\right]$$

where *T*_*c*_ and *T*_*h*_ is the temperature at cold and warm side respectively, Z *T*_*M*_ represents the materials optimum figure of merits among the cold and hot temperature end. Current research in thermoelectric devices is based on power generation that utilizes Seebeck effect, and cooling is achieved through Peltier effect. Very small/miniature version of thermoelectric device has more demand for cooling of several electronic circuit that brings in fabrication of thin film-based devices to meet the need for integrated circuit, and optical fiber communications technology^28,29^. Further, by lowering the dimensionality, materials properties such as S, σ, and κ can be controlled by tuning the lengthscale^26^.

The most popular design of thermoelectric modules used for waste heat harvesting are either planar or vertical style. Planar modules helps to build up the temperature gradient in the plane whereas the temperature difference can generated across the film thickness of the thermoelectric materials^30^. The formation of electrical contacts for planar type is simple compared to vertical type, and the device fabricated using a very low thermal conductivity substrate favor to create huge temperature difference in planar type module for optimum performance^30^. The device fabrication is cost effective as compared to other expensive processes where etching, photoresist and MEMS technologies are required. These limitations can be overcome by employing self-aligned shadow masking technique where the film deposition and TE modules are made in three stages: first one for negative semiconducting plane, second one for positive semiconducting plane, whereas third one is the electrical contacts that can connect both plane. The high vacuum and high substrate temperature favor to grow good quality film which is of prime importance to obtain the higher thermo-electric figure of merit^29^.

**Figure 1:**
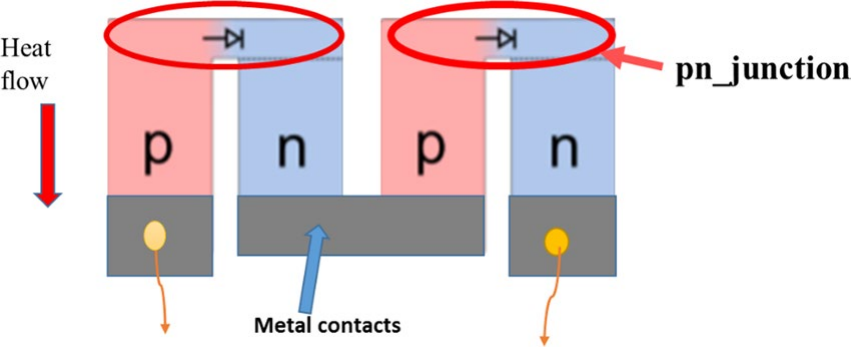
Schematic of pn-junction thermoelectric generator.

The development of temperature gradient for thin films structure is higher than the bulk counterpart module during the operation at same thermal condition, therefore, few layer thick films are ideal since larger temperature difference is required for high efficiency thermoelectric device^26,30,31^. The planar thin film module device design plays an important role in improving the efficiency of the TE device. Thermoelectric generators are connected electrically in series and thermally in parallel, this means the contacts at the hot end and cold end is the main factor that determines the efficiency of the TE device^32^. The current design, where metal electrode bonding on hot side, causes mechanical stress and unavoidable temperature drops across the substrates causing drawbacks of thermoelectric generator (TEG)^33^. Additionally, the design causes mismatch of the coefficient of thermal expansion between the metal-semiconductor interface at high temperatures, mechanically induced failure, chemical instability and diffusion of atoms across the interfaces^34^. In this paper, the design is based on pn-junction TEG, where the p-type and n-type planner patterns are connected directly at the hot end forming a pn-junction, and electrical contacts on the cold side. The design will alleviate the issues mentioned above and generate electron-hole pairs in space charge region, thus, contributing to thermoelectric device efficiency^33^. Figure 1 shows the schematic of pn-junction TEG.

In this paper, a pn-junction thermoelectric device for waste heat recovery and cooling was fabricated by RF magnetron sputtering of MoS_2_ and WS_2_ films on glass substrate at substrate temperatures that gave their individual high thermoelectric transport properties.

## Experimental Procedures

### Thin film deposition

MoS_2_ and WS_2_ films were deposited by RF magnetron sputtering on glass, sapphire and silicon substrates at several substrate temperatures, RF power, and argon pressure. Molybdenum disulfide (MoS_2_) and tungsten disulfide (WS_2_) targets with purity of 99.9% pure with a diameter of 3 inches by 0.125 inches thick, and indium bonded to copper backing plate were made by Kurt J. Lesker company. For electrical properties measurement, chromium electrodes were deposited on glass, sapphire substrates via thermal evaporation. The temperature difference voltage measurement was performed in indigenously developed test apparatus in the laboratory.

### Structural properties

Structural properties of the films studied using Rigaku X-ray diffractometer, using copper *K*_*α*_ source. Surface morphology and domain distribution of the films were characterized using atomic force microscope in tapping mode. X-ray photoelectron spectroscopy analysis of the film was performed to identify the chemical/oxidation state of the film. Vibrational modes were determined using HR Raman spectroscopy.

### Device fabrication

**Figure 2:**
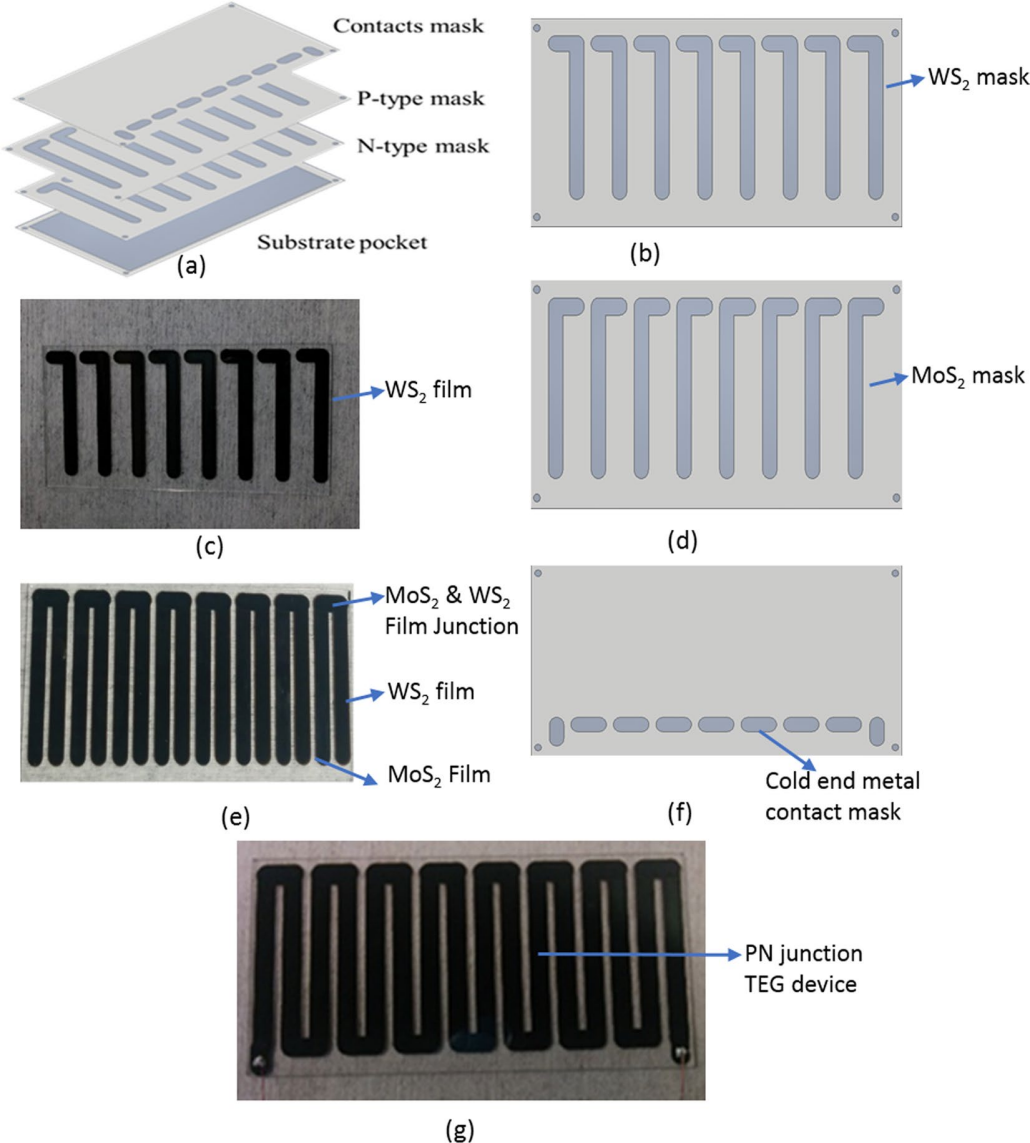
Schematic for step by step fabrication of thermoelectric pn junction device (**a**) Successive arrangement of different shadow mask for various materials deposited during device design. (**b**) WS_2_ leg mask set up on to the substrate prior to film deposition. (**c**) WS_2_ planar film considered as n-leg after deposition. (**d**) Arrangement of MoS_2_ mask for p-type planar leg onto the predisposed WS_2_ film. (**e**) Fabrication of MoS_2_ and WS_2_ film junction usually considered as hot end. (**f**) Alignment of mask for metal deposition to connect the other end of the both MoS_2_ and WS_2_ film. (**g**) PN junction based planar thermoelectric device structure.

Figure 2a illustrates a step by step procedure to fabricate the thermoelectric device using steel shadow mask and substrate pocket.

All the masks were designed using Autodesk inventor 2017 software, and steel were cut using expert mill at local workshop, Norfolk technical center, Norfolk, Virginia. Different masks are prepared with specific aperture dimensions to specify the profile of the thermoelectric legs and their metallic contacts for electrical measurement. We also focused the proper alignment of the mask during the device fabrication by securely tighten the four corner screw of 0.125 cm radius. The size of both semiconducting planner leg dimensions were 3.2 cm long and 0.317 cm wide. Whereas for proper electrical contact between two planar legs, the size of the metal contacts are 0.317 cm and 0.785 cm on its width and length, respectively, as shown in Fig. 2e,f. The individual planar patterns devices were placed 0.15 cm apart. The size of our entire thermoelectric structure with multiple planar legs was 7.5 cm × 3.6 cm^35^. Both MoS_2_ and WS_2_ films are deposited at the substrate temperature of 450 °C and annealed at 600 °C in Argon environments for 1 hour. The beginning step start with WS_2_ semiconducting planar leg of 700 nm thick, as a n-type material using the appropriate designed mask as shown in Fig. 2c, and cool it to room temperature prior to the deposition of p- type of leg. The second step of device design is to deposit the p-type of semiconducting leg consisting of 700 nm of MoS_2_ layer (Fig. 2e). At the end the metallic electrode of 70 nm chromium was deposited to interconnect the planar legs during electrical measurement (Fig. 2f). The final device was made out of 8 pairs of MoS_2_ and WS_2_ planar pattern to form the pn junction, usually exposed to the higher temperature end, and the metal contact connecting end, was exposed to lower temperature region.

### Characterization of the TE device

**Figure 3:**
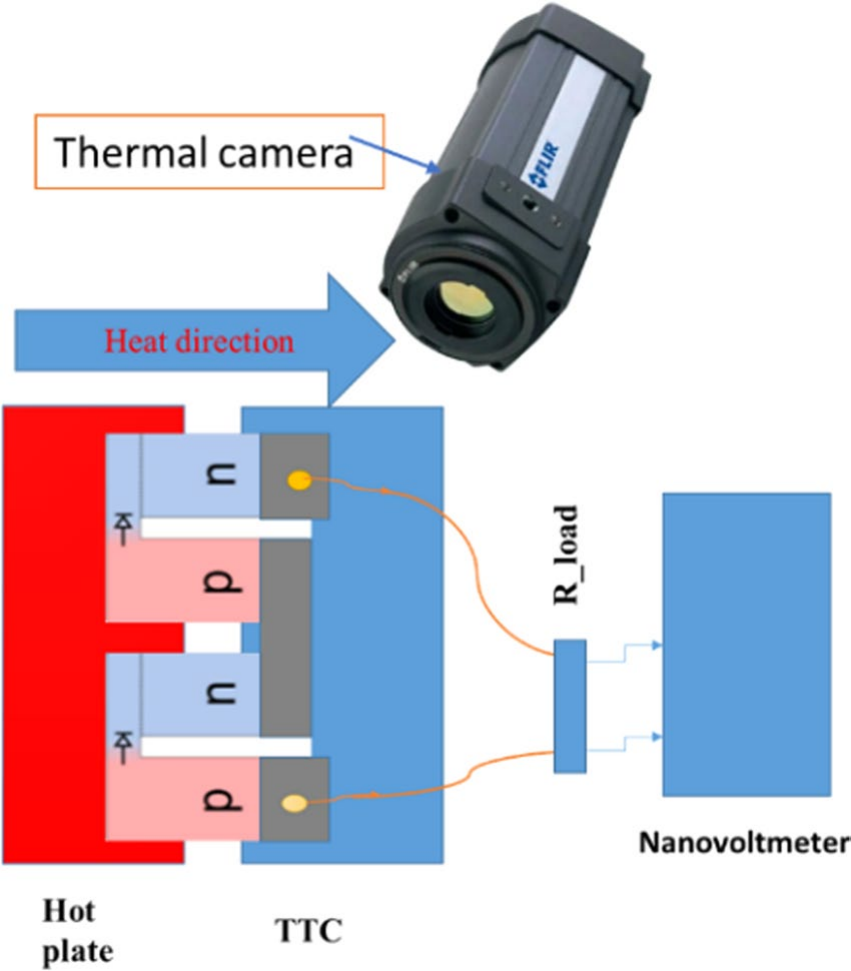
Hot plate and TTC Device testing set-up.

The schematic set up that was used to measure the performance of pn-junction TEG device was made in the lab, as shown in Fig. 3. The apparatus involved for the measurement were hot plate, thermoelectric temperature control system, known resistor load, Keithley 2182 A nanovoltmeter, and forward looking infrared (FLIR A320) thermal camera. The TE device was placed in between the hot plate and a temperature control system, thermal grease was applied to ensure good thermal contact between the device and hot plate, and thermoelectric temperature control (TTC). During the measurement, the temperature of the hot plate was increased gradually while maintaining the cold side at 20 °C using TTC. The device was connected in series with a known resistance R_L_, and with the nanovoltmeter, which is configured to measure output voltage in the circuit. The voltage readings were taken at every temperature increment of a hot plate from 25.4 °C to 295 °C. The temperature of the cold region increased to 62 °C due to heat diffusion from hot to cold region.

## Results and Discussions

**Figure 4:**
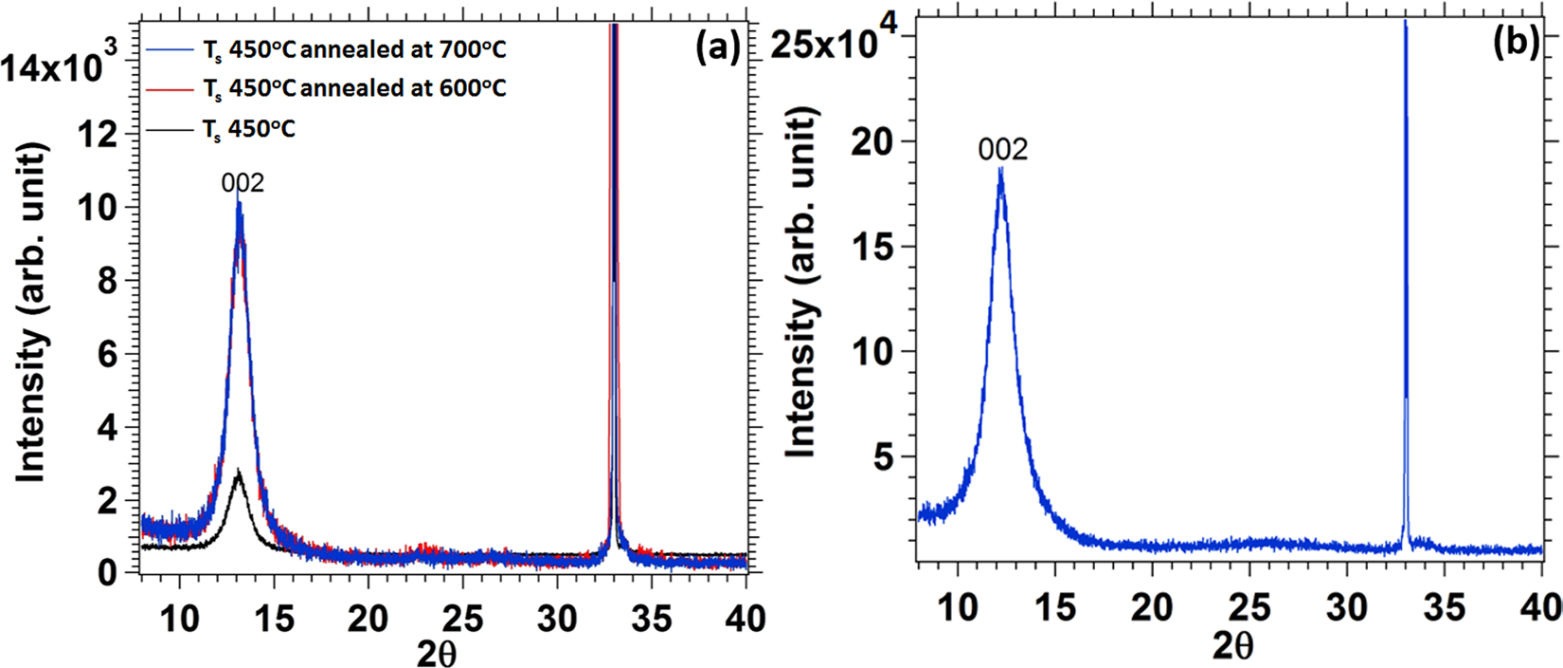
X-Ray diffraction graph of MoS_2_ film deposited at 450 °C and annealed at 600 °C and 700 °C for 1 hour at Ar2 environment and (b) X-Ray diffraction graph of WS_2_.

To study the crystalline quality of the films, X-ray diffraction was used. MoS_2_ deposited by RF sputtering at *T*_*s*_ = 450 °C revealed a pattern of a highly crystalline films with a strong orientation at 2θ = 13.6° that represents (002) MoS_2_, as shown in Fig. 4a. The peak at (002) shows crystal orientation with their basal planes parallel to the substrate surface^36,37^. However, high temperature annealing of the MoS_2_ film at 600 °C and 700 °C (Ar_2_ environment) for 1 hour improves the crystallinity quality a lot with sharp rise of the intensity up to four orders of magnitude as compare to as grown sample (450 °C). Further increase in the annealing temperature does not change any peak intensity or crystallinity of the film, but the thickness of the film start expanding with increasing in annealing temperature. Similarly, XRD graph of WS_2_ Film shows a very strong crystal orientation of hexagonal (002) peak, as shown in Fig. 4b display. No other peaks are found in both films even at higher 2θ value except the Si substrate peak at 2θ = 33°.

**Figure 5:**
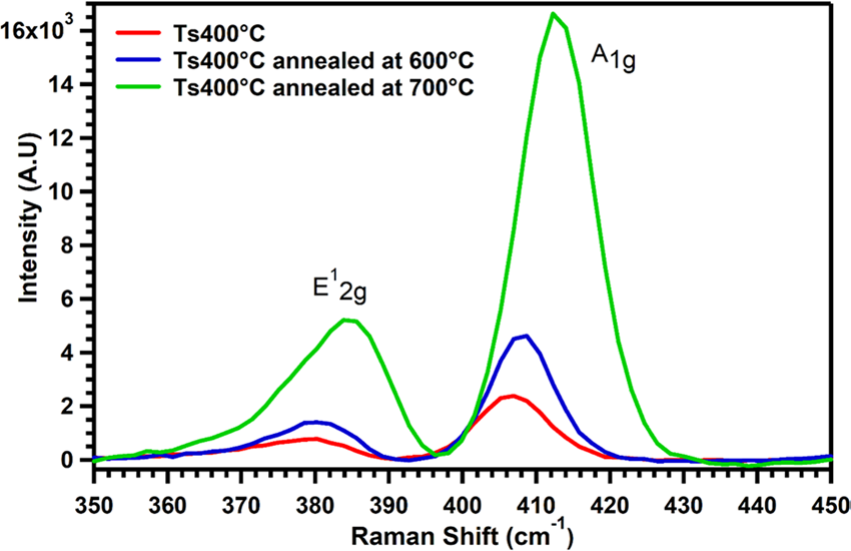
Raman spectra of MoS_2_ films on silicon (100). The curves in blue and green color display the sample annealed at different temperatures.

Raman spectroscopy of 150 nm MoS_2_ films deposited on Si (100) at *T*_*s*_ = 400 °C is displayed in Fig. 5. There are two distinct peaks of E^1^_2g_, and A_1g_ located at 380 cm^−1^ and 406 cm^−1^, corresponding to the characteristic feature of MoS_2_ film^38–43^. When the films were annealed at 600 °C and 700 °C in argon environment, we observe very interesting behavior and instead of shrinking, the film surprisingly start expanding from its original thickness and intensities of both vibrational modes increased as shown by the blue and the green curves. There is a clear redshift of E^1^_2g_ and A_1g_ vibrational modes, relative to the as grown red curve when the films were annealed at 700 °C. Annealing improves significantly the MoS_2_ crystal quality structure and causes the removal of surface contaminants and/or strain relief^40–42^. Similarly, we also observe that the increase in film thickness, due to annealing, also affects the characteristic of the Raman peaks and the intensity of the two peaks prominently increases after annealing at 700 °C. The increase in intensity of E^1^_2g_ mode is due to the increase in film thickness, however the increase in intensity of the A_1g_ mode is due to the change of force constant as a result of increasing number of layers, which has been studied elsewhere^39,43^. Furthermore, the microstructure of the sample also changes after annealing as shown in Supplementary Figs. (S1 and S2). The cross sectional view of the as grown and annealed MoS_2_ films are shown in Supplementary Fig. S3.

**Figure 6:**
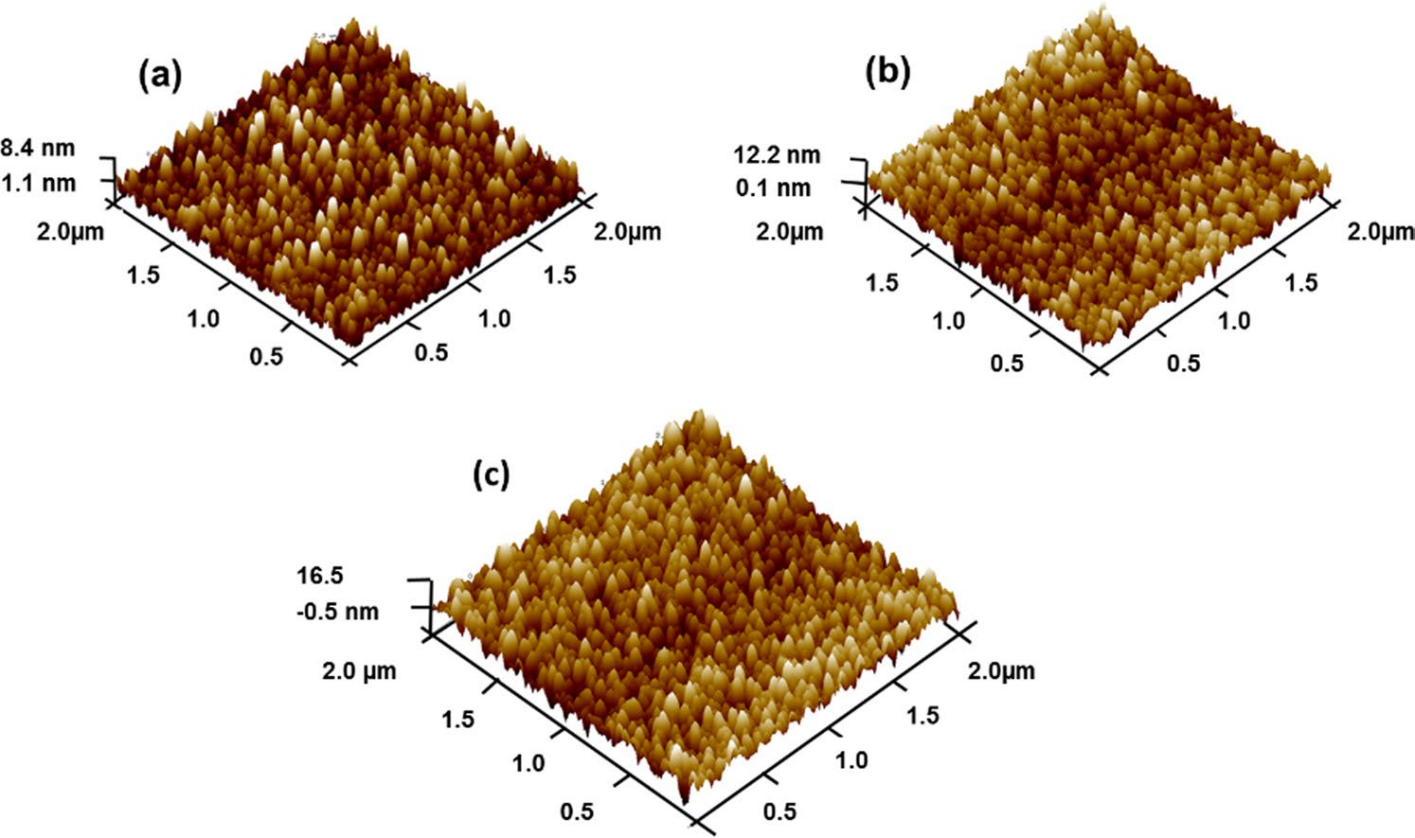
Displays the AFM 3D images of MoS_2_ film grown at (**a**) RT, (**b**) 150 °C and (**c**) 300 °C, respectively.

Atomic force microscopy of MoS_2_ films deposited at several substrate temperatures (*T*_*s*_) were studied and images are displayed in Fig. 6a–c. The films deposited on p-type Si (100) at *T*_*s*_ 300 °C displayed rms of 4.22 nm, and those deposited at *T*_*s*_ 150 °C displayed rms of 3.60 nm, and at room temperature deposition, the rms was 2.39 nm. AFM images of MoS_2_ thin films show that the surface topography and crystal size distribution increase with increasing substrate temperature (*T*_*s*_). The reduction in rms was due to reduction in crystallization of the films when the temperature was lowered. The crystal size plays a role in the film properties. The surface roughness of the films has opposite effect in electrical conductivity of the films, in some cases the roughness leads to a reduction in electrical conductivity^44^. The roughness and grain boundaries cause additional scattering centers for conduction electrons leading to a higher electrical resistivity^45^. Figure 6a–c display 3D AFM images of MoS_2_ films deposited on silicon.

**Figure 7:**
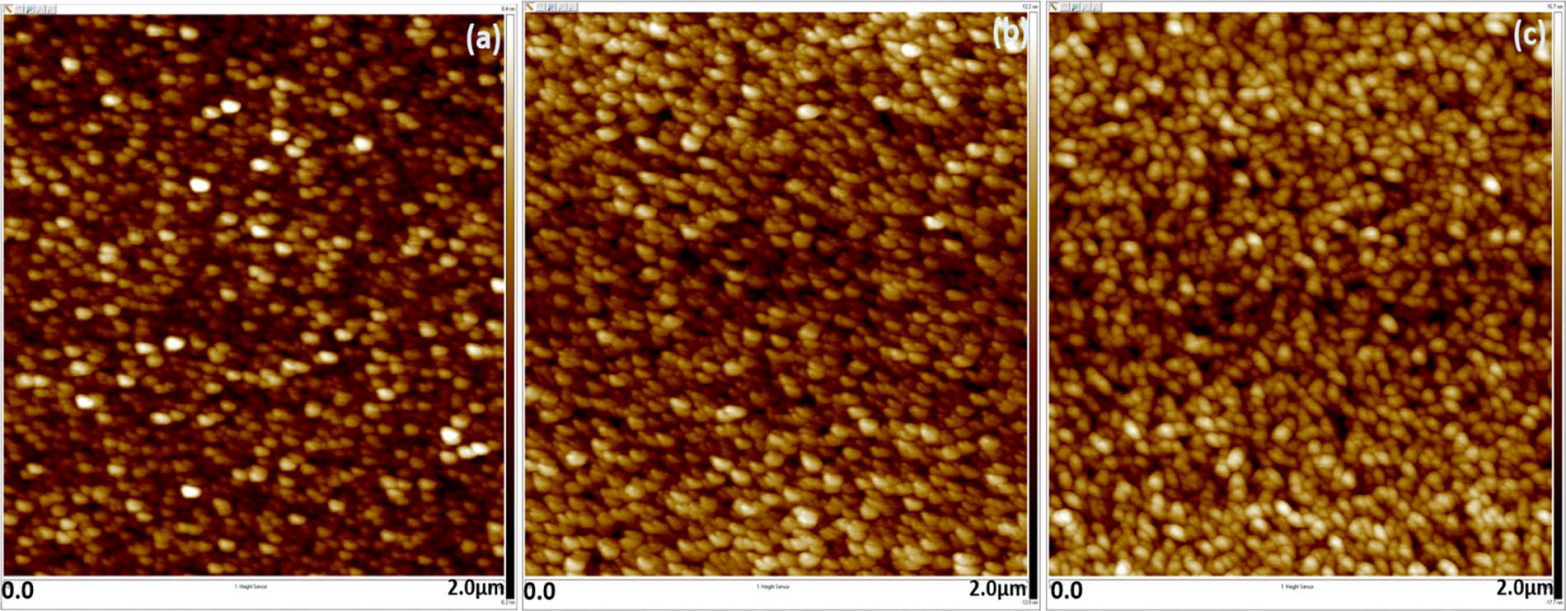
AFM 2D images of MoS_2_ film grown at (**a**) RT, (**b**) 150 °C and (**c**) 300 °C, respectively.

The 2D images of the MoS_2_ films deposited on silicon substrate at various temperature, are shown in Fig. 7a–c. The grains of the RT growth films are uniformly distributed on the surface of the sample with small amount of void present between the grains. However, the increase in growth temperature helps to develop larger grains of MoS_2_ and the grain growth helps to reduce the void present among the grains. Furthermore, increase in substrate temperature favors to obtain a densely-packed grains over the MoS_2_ film.

**Figure 8:**
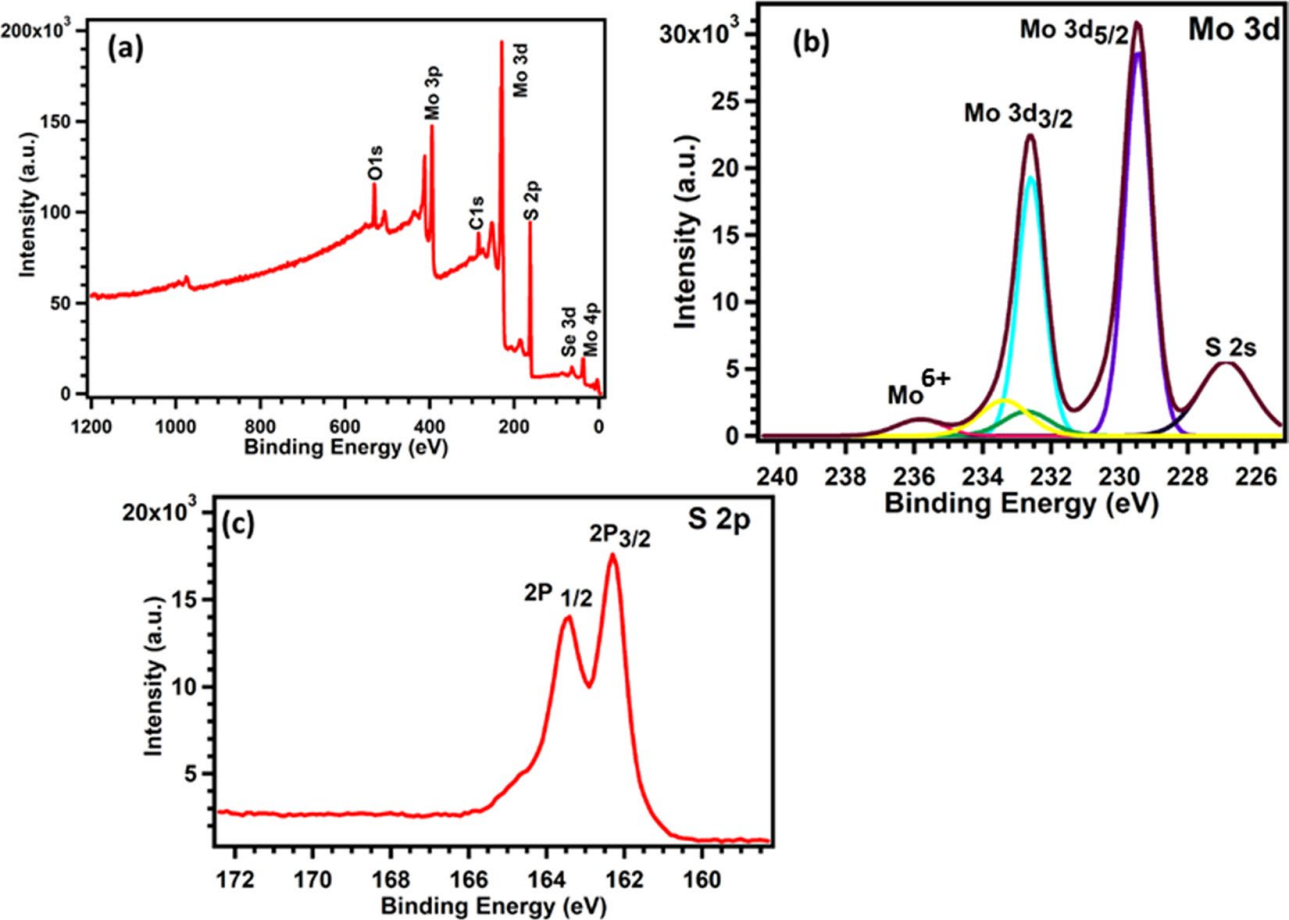
The X-ray photoelectron spectroscopy of MoS_2_ films: (**a**) XPS survey spectrum (**b**) High resolution XPS spectrum of Mo 3d peaks, and (**c**) High resolution XPS spectrum of the S 2p peaks levels.

XPS analysis were used to study the binding energies of Mo and S atoms in MoS_2_ Table 1 shows XPS elemental analysis of MoS_2_ films deposited on silicon substrate. Figure 8a shows wide scan of MoS_2_ films. Binding energies of Mo 3d and S 2p in electrons volts are shown in Fig. 8b,c, respectively. Two distinct peaks at 229.4 eV and 232.7 eV are attributed to doublet; Mo 3d_5/2_, and Mo 3d_3/2_, respectively^41–43^. However, a small peak is observed at 236 eV is corresponding to Mo^6+^ obtained from the formation of small amount MoO_3_ in the MoS_2_ film surface due to exposure to outside environment for longer period of time^38^. The peaks corresponding to S 2p state are shown in Fig. 8c, the two peaks located at 162.5 eV and 163.3 eV correspond to S 2p _3/2_ and S 2p_1/2_, respectively. They represent orbital divalent sulfides ions (S^2−^)^42–46^. The observed peak positions of spin-orbit coupled Mo 3d and S 2p states are in agreement with the literature and confirms the synthesis of MoS_2_ thin film^38,43^.

**Table 1:** Elemental analysis of MoS_2_ films.

Peak	Type	Position BE(eV)	FWHM (eV)	Raw Area (cps eV)	RSF	Atomic Mass	Atomic Conc. %	Mass Conc. %
C 1 s	Reg	284.403	1.459	3632.4	0.278	12.011	14.61	4.21
O 1 s	Reg	530.803	1.977	9066.5	0.78	15.999	12.01	4.61
Si 2p	Reg	107.303	10.8	0	0.328	28.086	0	0
Mo 3d	Reg	229.403	0.894	66103.1	3.321	95.922	22.63	52.11
S 2p	Reg	162.303	1.919	29287	0.668	32.065	50.75	39.07

**Figure 9:**
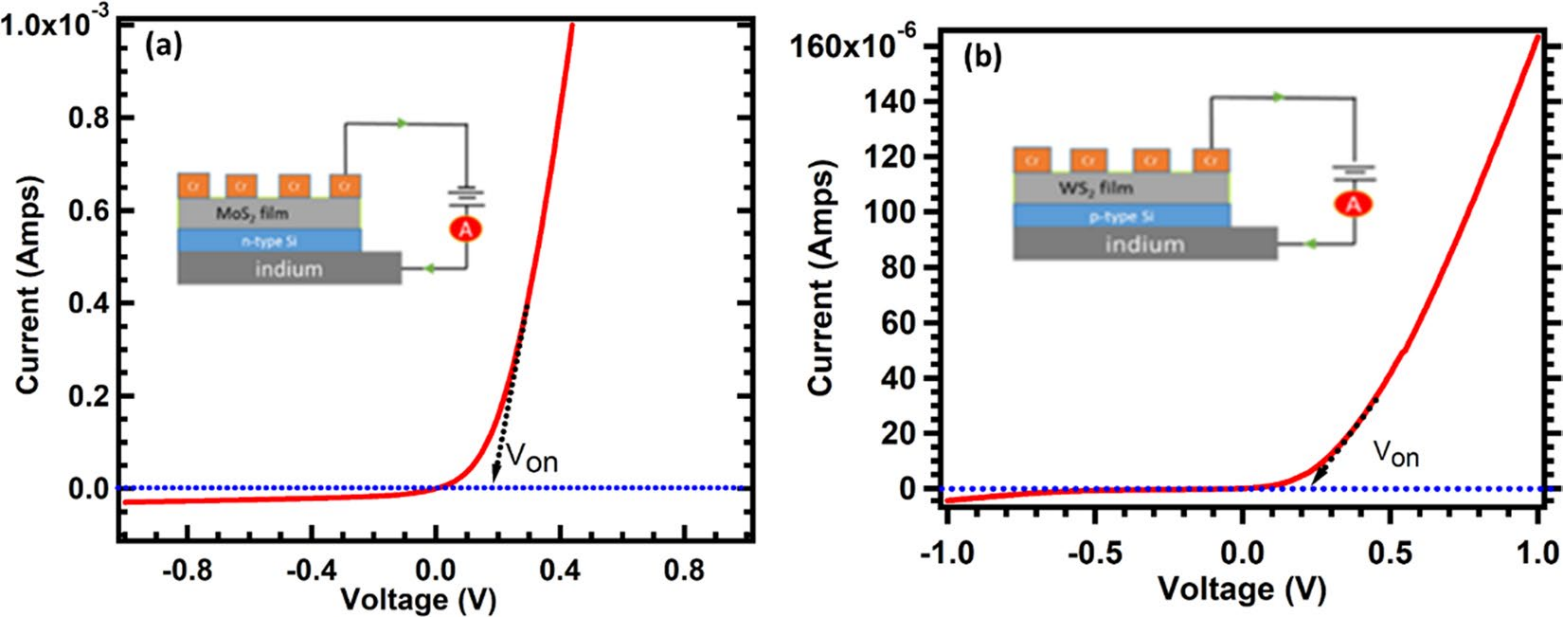
(**a**,**b**) Display the I-V characteristics of MoS_2_ and WS_2_, respectively. The inset shows the schematic illustration setup of the electrical measurement of the MoS_2_/n-type silicon, and indium as the bottom contact.

I-V characteristics of MoS_2_ and WS_2_ were measured at room temperature and shown in Fig. 9a,b. The inset represents the schematic of I-V measurement of the device. MoS_2_ was deposited on n-type Si, while WS_2_ on p-type Si with substrate temperature of 450 °C and annealed them at 600 °C in Argon environments for one hour. The thickness of the both films are 700 nm. Both had indium bottom contact and top chromium contacts, as shown in the insets. Both curves display rectifying characteristics at a turn on voltage (V_on_) of 0.2 V where the current starts to increase infinitely^43,47,48^.

**Figure 10:**
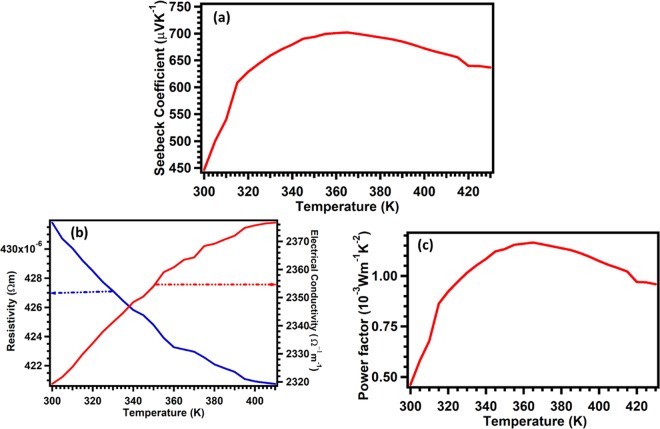
Thermoelectric transport properties of MoS_2_. (**a**–**c**) Display Seebeck coefficient, electrical measurement, and power factor of MoS_2_, respectively.

Seebeck coefficient of MoS_2_ was performed from 300 K to 430 K at a temperature interval of 5 K using digital Seebeck controller measurement set up (Make-MMR technologies, model- SB1000). During the measurement the temperature difference (ΔT), between the hot and cold regions, was between 4 to 5 K. Figure 10a, displays the Seebeck coefficient temperature dependence. The positive Seebeck coefficient represents majority of hole carriers which are present in MoS_2_ films typical for p-type semiconductor material^49–51^. Temperature dependent resistivity of the films was performed using linear four-probe technique from room temperature to 420 K. During the measurement a fixed current of 1 mA was applied between the outer two probes using a Keithley 6220 precision current source. The voltage was measured using a Keithley 2182 A nanovoltmeter. Figure 10b displays the resistivity/conductivity of MoS_2_ films. The films display semiconducting behavior.

The power factor was calculated using the product of Seebeck coefficient squared and electrical conductivity (pf = *S*^2^*σ*) measured from RT to 430 K. The results display strong temperature dependence, where it increases with temperature until a maximum value of 1.15 × 10^−3^ Wm^−1^ K^−2^ is reached at 370 K, as shown in Fig. 10c below, before it started to decrease.

Thermal conductivity of MoS_2_ was measured at room temperature using time domain thermal reflectance (TDTR) at modulation frequency of 0.824–9.68 MHz. An Aluminum transducer layer of 88 nm was deposited on 850 nm thick MoS_2_ sample to serve as an energy absorber for the laser beams as well as temperature transducer for the probe beam^52^. The films deposited on glass substrates displays low cross-plane thermal conductivity of 0.07–0.5 W/mK at 300 K. The calculated figure of merit of highly crystalline MoS_2_ films, assuming the thermal conductivity is approximately isotropic, was approximately 0.27–1.98 at 300 K, as shown in Table 2. At the high end, this value is higher than the most efficient thermoelectric materials, such as Bi_2_Te_3_ with its value approximately <1 at room temperature^53^. The reduction of thermal conductivity is due to the surface roughness that interacts with a broadband spectrum of phonons resulting in decreased thermal conductivity due to frequency dependent scattering^45,54–56^. Additionally, the effect of low thermal conductivity is attributed to grain size and grain boundaries that act as phonon scattering centers^57^.

**Table 2:** Calculated figure merit.

Seebeck Coeff. (μVK-1)	Electrical Conductivity (Ω^−1^ m^−1^)	Thermal Conductivity (Wm^−1^ K^−1^)	Temperature (K)	Figure of merit
466.34	2320	0.07–0.50	300	0.27–1.98

**Figure 11:**
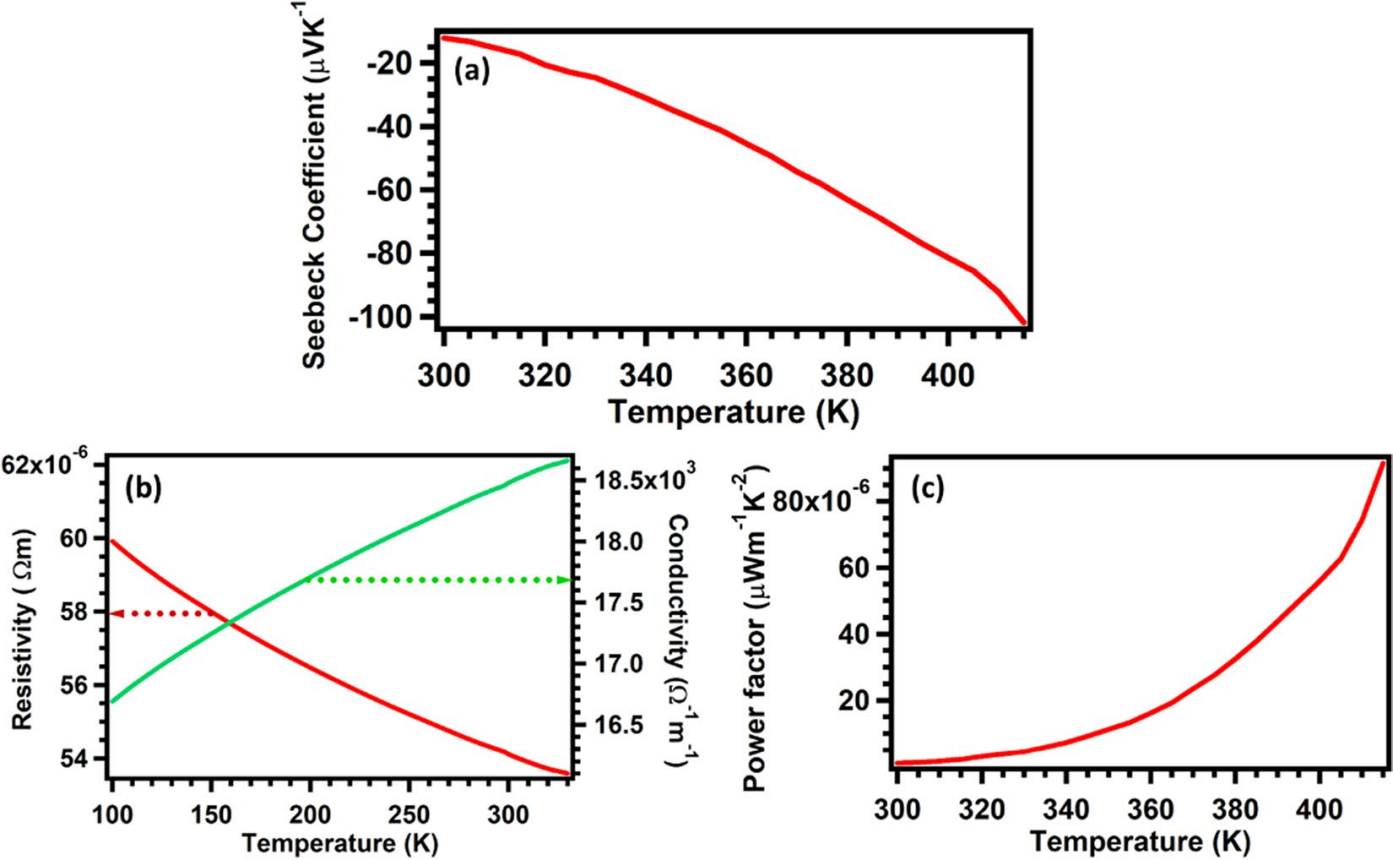
Thermoelectric transport properties of WS_2_. (**a**–**c**) Display Seebeck coefficient, resistivity and power factor of WS_2_ films.

Seebeck coefficient of WS_2_ was measured from 300 K to 420 K, as shown in Fig. 11a. The absolute value of Seebeck coefficient increases with temperature, exhibiting negative thermo-power, implying that electrons were the majority carriers in the WS_2_ films, which is normal for n-type material. Resistivity measurements exhibits semiconductor behavior, as shown in Fig. 11b, while the power factor increases with temperature, Fig. 11c.

**Figure 12:**
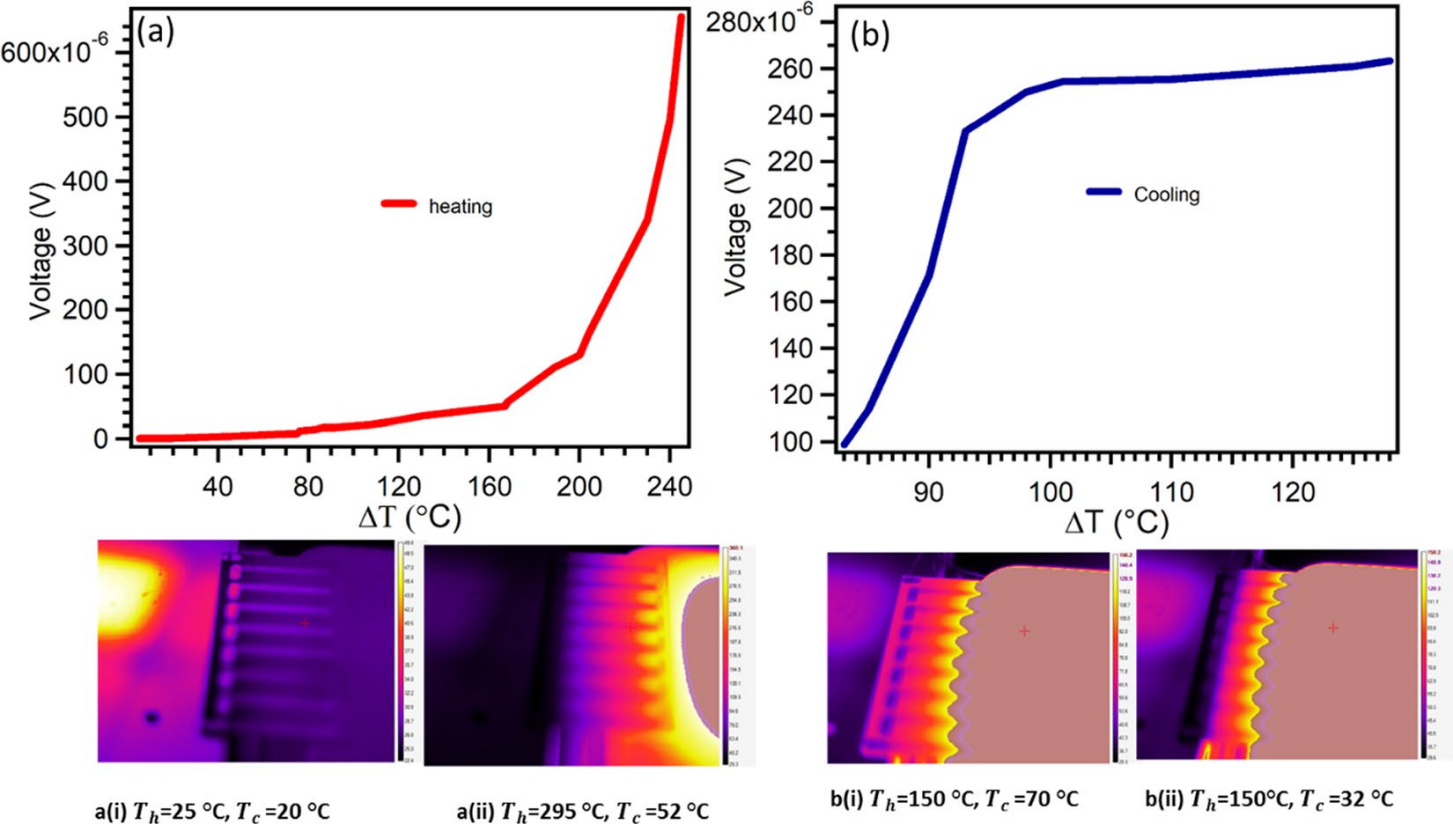
Voltage measurement. (**a**,**b**) Heating and cooling of TE device (MoS_2_- WS_2_), respectively.

Figure 12a,b display temperature gradient versus output voltage of a thin film pn-junction TE device (MoS_2_ (p-type) and WS_2_ (n-type)) for both heating and cooling. Voltage increases with increase in temperature gradient. For the device heating experiment, TTC was set at 20 °C whereas the hot plate temperature was set to start at 25 °C. The temperature of the hot side was increased gradually until 295 °C, as seen by thermal camera images of Fig. 12a (i, and ii). During cooling, the hot plate temperature was maintained at 150 °C, while the TTC was set to start decreasing from 70 °C to 32 °C, as shown in thermal camera image of Fig. 12b (i, and ii). The cooling testing shows that the planar thin film device is actively used for cooling up to ΔT of 95 °C, as seen by steeper curve in Fig. 12b. Device heating voltage of 0.7 mV was achieved when temperature gradient was 240 °C. Whereas cooling produced a voltage of 0.27 mV at a temperature gradient of 118 °C.

## Discussion

We have successfully demonstrated large-area highly crystalline MoS_2_ thin films deposited on different substrates via RF magnetron sputtering technique. XRD revealed highly oriented (001) films and AFM display shows the homogenous distribution of MoS_2_ grain on the Si substrate. MoS_2_ films revealed ultralow thermal conductivity of 0.07 W/mK at 300 K, which is a good candidate for thermoelectric applications. Additionally, pn junction TEG based on MoS_2_ (p-type) and WS_2_ (n-type) planner structure demonstrates superior energy harvesting and cooling performance compared to convention TEG, where metal contacts are done on both hot region and cold region. The crystalline quality of the films was improved via annealing at 600 °C and 700 °C in an argon environment. This produced increased intensity and red shifted of $\,{E}_{2g}^{1}$ and ${A}_{1g}$ Raman peaks.

## Conclusion

We have studied thermoelectric transport properties of highly crystalline MoS_2_ films as well as WS_2_ films deposited by RF magnetron sputtering for high-efficient thermal energy harvesting applications. AFM images display an increase in rms when MoS_2_ films were grown at higher substrate temperatures. Surface roughness of the films has opposite effect in electrical conductivity and thermal properties of the films. Roughness and grain boundaries leads to an increase in resistivity of the films. MoS_2_ films indicate low thermal conductivity and a Seebeck coefficient of 446.342 µV/K, suggesting a figure of merit of up to 1.98 at 300 K. Also the thermoelectric generator fabricated based on pn-junction at the hot end and chromium contacts at the cold end demonstrates superior efficiency performance at high temperature gradients, due to generation of electrons–holes at the depletion region of the pn-junction. In addition, the pn-junction makes the device more robust at higher temperatures by withstanding thermal-mechanical stress, alloying, inter diffusion of atoms, and mismatch of coefficients of thermal expansion between the materials in contact at the hot side. Device heating voltage of 0.7 mV was realized at a temperature gradient of 240 °C. Whereas cooling produced a voltage of 0.27 mV at a temperature gradient of 118 °C. These promising results suggest that the material can potential be used for moderate waste heat harvesting.

## Methods

### The film growth

Both WS_2_ and MoS_2_ thin films were grown from the target materials by physical vapor deposition technique using radio frequency magnetron sputtering on to the various substrates. Prior to the deposition, all the substrates were cleaned thoroughly by dipping them to different solvent such as acetone, methanol and isopropanol for 5 minutes each solution in ultrasonic bath. Then the substrate were cleaned using deionized water and followed by dried them in nitrogen gun. High purity targets of MoS_2_ and WS_2_ (99.99% purity) were used as source materials and cleaned them in Ar2 plasma environment for 20 minutes to remove any oxide/contaminants from the target surface. All the substrate were transferred to high vacuum 1.6 × 10^−6^ Torr deposition chamber for the optimum thickness of MoS_2_ and WS_2_ thin film grown. The growth parameter for MoS_2_ films were 2.3 mTorr Ar_2_ pressure, 150 W plasma power. Whereas WS_2_ films were grown at 5mTorr Ar_2_ pressure, 150 W plasma power. All the films are grown for different period of time from 10–45 minutes to obtain various film thickness. The films were annealed at 600 °C and 700 °C under argon atmosphere at a flow rate of 50 sccm to improve the crystallinity of the films^35^.

### Fabrication of pn-junction TE device

The PN junction based thermoelectric devices were fabricated using different shadow mask and proper alignment of the planar leg. All the step by step masks were designed by cutting thin sheet of steel using local workshop. The films were grown at high vacuum and much above room temperature and precaution were taken to avoid contamination. The devices was fabricated on glass substrate; first WS_2_ films were deposited followed by MoS_2_ films to make pn junction at one end of the structure which is considered as a hot end. Other end of the WS_2_ and MoS_2_ legs were connected using 60 nm of Cr metal contact using thermal evaporation technique^35^.

### Characterization details of MoS_2_ and WS_2_ films

Synthesis of MoS_2_ films were analyzed by Raman spectroscopy (LabRAM HR Evolution, 532 nm laser) with a spot size of 1 µm and acquisition time of 300 seconds. A silicon substrate with a Raman peak of 520.6 *cm*^−1^ offset: 478 and coefficient of 6.07 × 10^−3^ was used to calibrate the instrument. The crystallinity of the films was measured by X-ray diffractometer (XRD, Rigaku) using Cu *K*_*α*_ X-ray source operated at 20 kV, and 40 mA. Photoelectron spectroscopy (XPS) was used to study the elemental analysis of Mo and S atoms. To study morphology and grain size atomic force microscopy (AFM) (Veeco, Dimension Icon Scan Asyst) was used. Thermoelectric properties and Seebeck coefficient was measured using MMR technologies. Resistivity/conductivity was measured using standard four-probe technique (APD cryogenics). IV measurements were measured using Keithley 4200-SCS (semiconductor characterization system). Total thermal conductivity was measured using TDTR, operated at modulation frequency of 0.824–9.68 MHz. For temperature dependent electrical study, the samples are inserted into the cryostat and standard linear four-probe technique was used for data acquisition^35^.

## Supplementary information

Supplementary information
