# Supplementary material for: A thin film efficient pn-junction thermoelectric device fabricated by self-align shadow mask
Source: Sci Rep. 2020 Jan 23;10:1067. doi: 10.1038/s41598-020-57991-y (PMC6978454; doi:10.1038/s41598-020-57991-y)
Supplement: Supplementary file 1 — Supplementary information [file 41598_2020_57991_MOESM1_ESM.docx]

**Supplementary information**

**A thin film efficient pn-junction thermoelectric device fabricated by self-align shadow mask**

*^*a^***Gilbert Kogo,** *^a^***Bo Xiao, and** *^a^***Samuel Danquah,** *^a^***Harold Lee,** *^a^***Julien Niyogushima, ^a^Kelsea Yarbrough,** *^b^***Aaditya Candadai,** *^b^***Amy Marconnet, ****^a^***Sangram K Pradhan,**  *^a,c^***Bahoura Messaoud**

*^a^Center for Materials research, Norfolk State University, Norfolk, Virginia 23504, USA*

*^b^Mechanical Engineering Purdue University, West Lafayette, In 47907*, *USA*

*^c^Department of Engineering, Norfolk State University, Norfolk, Virginia 23504, USA*


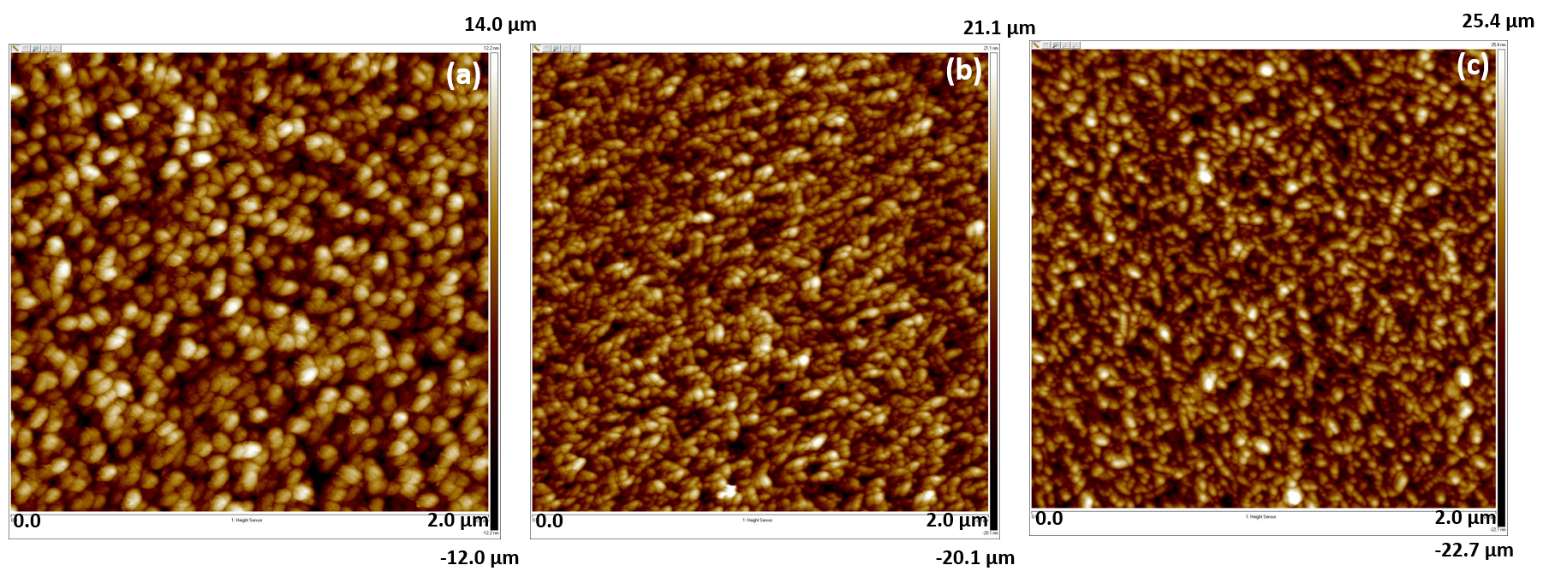


**Figure S1**. AFM 2D images of MoS_2_ film (**a**) grown at 400^o^C, (**b**) annealed at 600 °C and (**c**) annealed at 700°C.

The atomic force microscopy 2D images of the as grown MoS_2_ film deposited on silicon at the substrate temperature of T_s_=400 ^o^C, are shown in figure 1a. The grains are well distributed on the sample surface with surface roughness value of 3.28 nm. The corresponding 2D image of the sample is shown in Fig 2a. After annealing the sample at 600 ^o^C for 1 hour in Ar_2_ environment (figure 1b), we observed the formation of more grains with very small in size on the surface of the sample which helps to improve the crystalline quality of the sample. The distribution of grains on the sample surface is seems to very compact in nature and it enhance the surface roughness value of the sample to 5.55 nm. Hence, increase in annealing temperature favors to obtain a densely-packed grains over the MoS_2_ film. The corresponding 3D image of the sample is shown in figure 2b. Further increase in annealing temperature (700 ^o^C for one hour) does not affect any substantial change of surface morphology a lot as shown in figure 1c but only favor to increase the surface roughness of the sample to 6.09 nm. The corresponding 3D AFM image is shown in figure 2C.


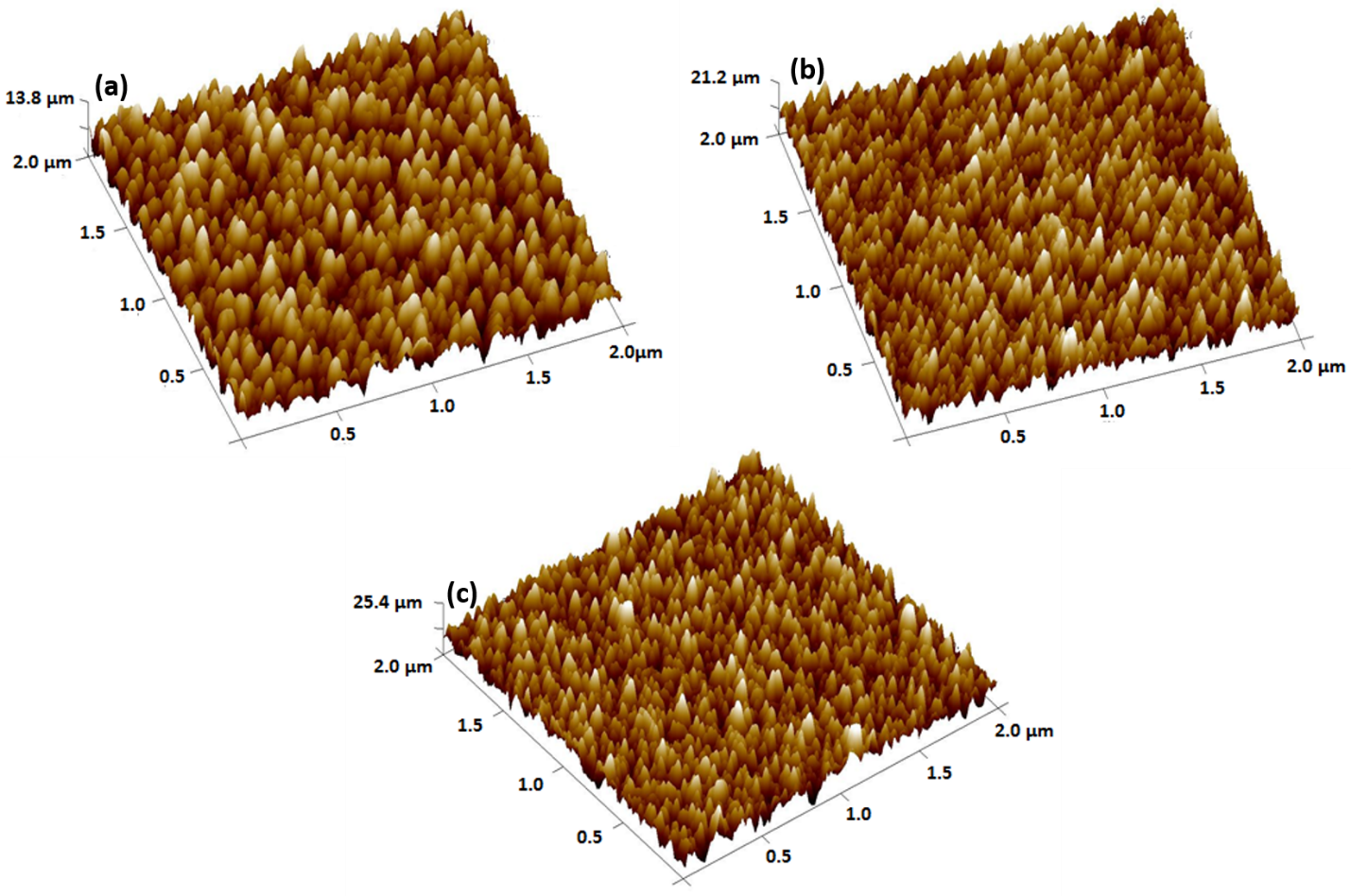


**Figure S2**. AFM 3D images of MoS_2_ film (**a**) grown at 400 ^o^C, (**b**) annealed at 600 °C and (**c**) annealed at 700°C.

The cross sectional FESE image of the MoS2 film grown at 400 ^o^C and annealed at 700 ^o^C are shown in figure S3. The sample shows the expansion behavior during annealing as shown in figure (S3 b).


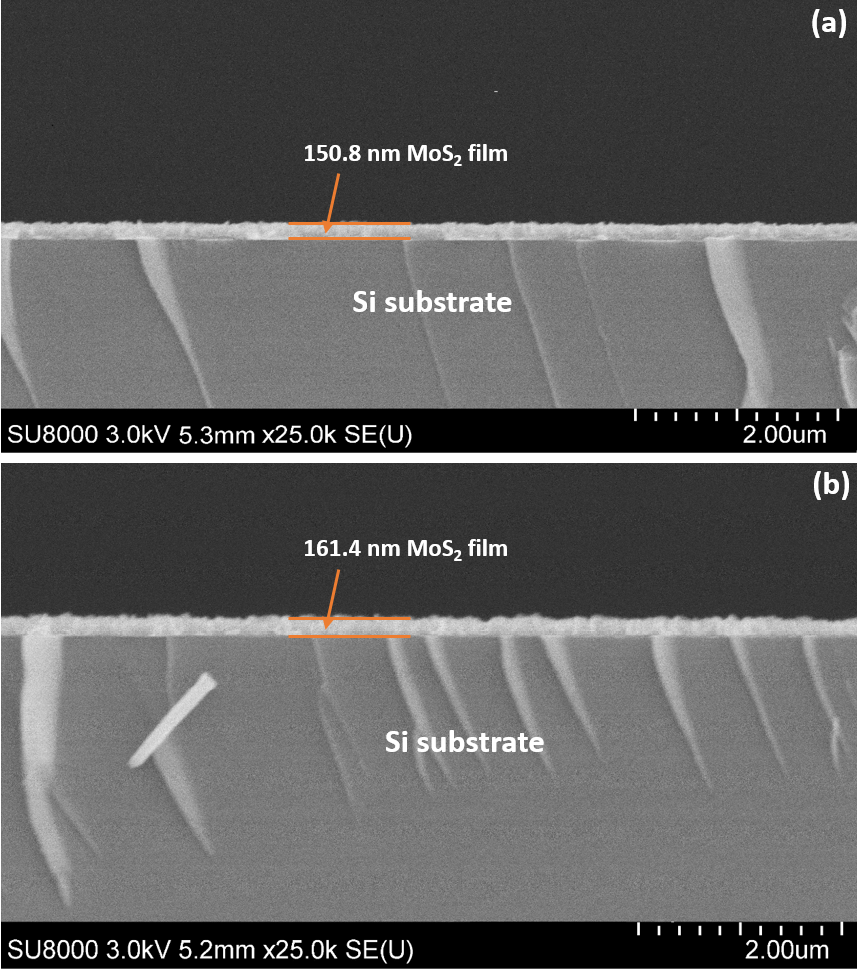


**Figure S3**. Cross sectional image of MoS_2_ film (**a**) grown at 400 ^o^C, (**b**) annealed at 700 °C.
